# Broad-Spectrum Antibody-Based Immunochromatographic Strip Assay for Rapid Screening of Bisphenol A Diglycidyl Ether and Its Derivatives in Canned Foods

**DOI:** 10.3390/molecules29010013

**Published:** 2023-12-19

**Authors:** Chundi Yu, Jinnuo Hu, Wei Wu, Yongfei Zhou, Can Zhang, Qingli Yang

**Affiliations:** 1 College of Food Science and Engineering, Qingdao Agricultural University, Qingdao 266109, China; naturalyu@qau.edu.cn (C.Y.); wuweiouc@126.com (W.W.); 2 School of Food and Biological Engineering, Jiangsu University, Zhenjiang 212013, China; qdxdhou@qau.edu.cn (J.H.); tangjuan@qau.edu.cn (Y.Z.); 3 Academy of Dongying Efficient Agricultural Technology and Industry on Saline and Alkaline Land in Collaboration with Qingdao Agricultural University, Qingdao 266109, China; 4 Qingdao Institute of Special Food, Qingdao 266109, China

**Keywords:** bisphenol A diglycidyl ether, derivatives, broad-spectrum polyclonal antibodies, gold nanoparticles, immunochromatographic strip assay, canned food

## Abstract

Bisphenol A diglycidyl ether (BADGE) is widely present in the inner coating of metal food cans, from which it can migrate into food and generate harmful derivatives during storage, such as bisphenol A (2,3-dihydroxypropyl) glycidyl ether, bisphenol A (3-chloro-2-hydroxypropyl) glycidyl ether, and bisphenol A (3-chloro-2-hydroxypropyl) (2,3-dihydroxypropyl) glycidyl ether. Here, a gold-nanoparticle-based immunochromatographic strip assay based on a broad-spectrum polyclonal antibody was developed for the simultaneous detection of BADGE and its derivatives, which could be accomplished within 15 min. The quantitative analysis of the visualization results was performed using Adobe Photoshop CC 2021, and the detection limit, defined as the concentration causing 15% inhibition, was 0.97 ng/mL. The recoveries of BADGE and its derivatives at various spiking levels in canned food samples ranged from 79.86% to 93.81%. The detection results of the proposed immunochromatographic strip assay were validated via high-performance liquid chromatography, showing a good correlation coefficient (R^2^ = 0.9580).

## 1. Introduction

Bisphenol A diglycidyl ether (2,2-bis(4-glycidyloxyphenyl) propane, BADGE) is a condensation product of bisphenol A (BPA) and epichlorohydrin. It can be used as an additive in polyester fibers and as a hydrochloric acid scrubber. In particular, BADGE is often utilized to remove the hydrochloric acid in the organosol resins used as inner coatings of metal food cans [1,2,3], where it can remain if the chemical reaction is not complete during the coating manufacturing process [4,5] and migrate into food during processing and storage [6,7]. Due to the complexity of the food matrix, BADGE can react with acidic or greasy food via hydrolysis or chlorination, generating various derivatives, such as bisphenol A (2,3-dihydroxypropyl) glycidyl ether (BADGE·H_2_O), bisphenol A (3-chloro-2-hydroxypropyl) glycidyl ether (BADGE·HCl), and bisphenol A (3-chloro-2-hydroxypropyl) (2,3-dihydroxypropyl) glycidyl ether (BADGE·HCl·H_2_O) [8,9], which have been detected along with BADGE in canned foods such as canned seafood, canned meat products, and energy drinks [10,11]. BADGE may lead to abnormalities in the human endocrine, immune, and nervous systems and affect normal reproductive and genetic functions [12,13,14]. Therefore, European legislation has set specific migration limits for BADGE and its hydrolysis derivative BADGE·H_2_O at 9 mg/kg in foodstuffs and food simulants as well as for the hydrochloric derivatives BADGE·HCl and BADGE·HCl·H_2_O at 1 mg/kg [15].

Various analytical methods based on high-performance liquid chromatography–fluorescence detection (HPLC–FLD) [16,17,18], liquid chromatography–tandem mass spectrometry (LC-MS) [19,20,21], and gas chromatography–mass spectrometry (GC–MS) have been developed for the detection of bisphenol-dihydrate glycerol ether [22]. Although LC-MS and GC–MS have good accuracy and high sensitivity, they are expensive, complex, and require trained operators, which is not conducive to rapid on-site detection. In contrast, immunoassays are suitable for the simultaneous detection of a large quantity of samples and can meet daily monitoring requirements. The immunochromatographic strip assay, also known as lateral-flow immunoassay, is a combination of chromatography and immunoassay. It has gained attention as an alternative to enzyme-linked immunosorbent assay (ELISA). Immunochromatographic strip test is considered to be a useful tool for the rapid screening of food and raw materials. It is in demand for rapid and point-of-care testing in all parts of the food-processing chain “from farm to fork” [23,24].

Gold nanoparticles are commonly used as detector reagents in lateral-flow immunochromatography for the visualization of signals. In particular, gold nanoparticle (AuNP-based immunochromatographic strip assays have been widely used for detecting hazardous macromolecular and small-molecule substances [25,26,27,28,29,30,31].

To the best of our knowledge, although the production of monoclonal antibodies that can recognize BADGE and the establishment of an ELISA method for detecting BADGE in lake water have been previously reported [32], broad-spectrum polyclonal antibodies that can simultaneously recognize BADGE and its derivatives using an immunochromatographic strip assay have not yet been described.

Here, we developed an immunochromatographic strip assay using AuNPs and a broad-spectrum polyclonal antibody to simultaneously detect BADGE and its derivatives. The visualized results were quantitatively analyzed using Adobe Photoshop CC software. Canned foods were selected as samples, and HPLC was used as the analysis method to evaluate the proposed immunochromatographic strip assay, which is a convenient tool for the fast and efficient detection of BADGE and its derivatives.

## 2. Results

### 2.1. Screening of Broad-Spectrum Antibodies

**Figure 1:**
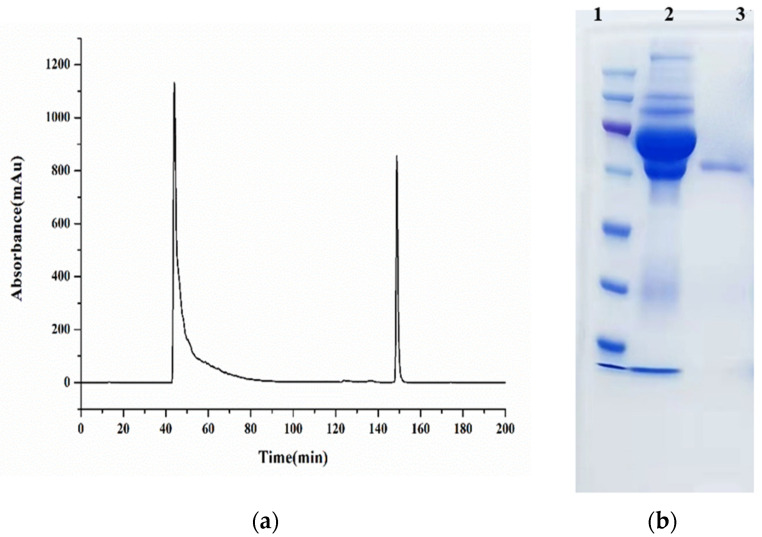
(**a**) The antiserum purified via protein A-Sepharose 4B affinity chromatography. (**b**) The results of SDS-PAGE: 1: marker; 2: antiserum; 3: purified antibody.

Standard solutions of BADGE derivatives were freshly prepared before analysis. The concentration causing 50% inhibition (IC_50_) values were determined, and the cross-reactivities were calculated using the indirect competitive ELISA (ic-ELISA) method. The recognition capability of selected antisera (PAb-1, PAb-2, PAB-3, and PAb-4) for BADGE derivatives was also investigated. As shown in Table 1, the cross-reactivities of antiserum PAb-1 with BADGE·HCl, BADGE·H_2_O, and BADGE·HCl·H_2_O were 79.6%, 175.8%, and 110.8%, respectively. Compared with other antisera, antiserum PAb-1 exhibited better recognition capability for BADGE and its derivatives; therefore, it was selected as a broad-spectrum recognition antiserum. PAb-1 was purified using protein A-Sepharose 4B affinity chromatography (Figure 1a), and the obtained antibody was characterized using SDS-PAGE (Figure 1b).

### 2.2. Optimization of AuNP-Labeled Antibody

**Figure 2:**
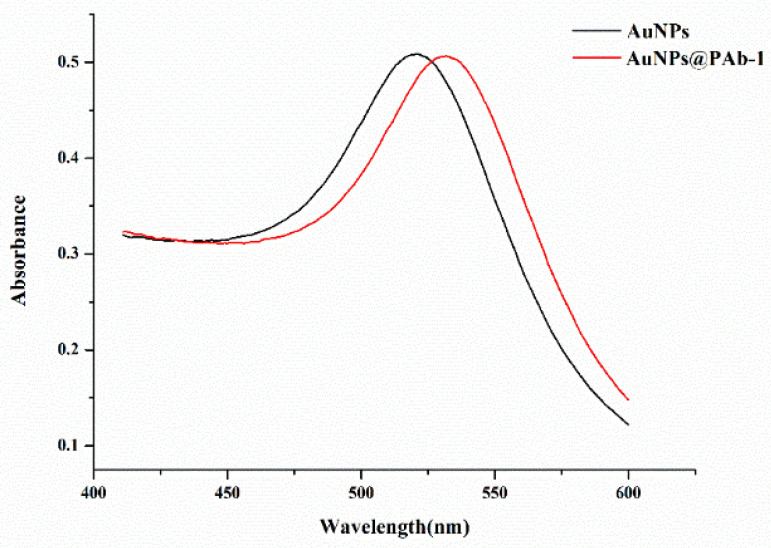
Wavelength scanning of AuNPs and AuNP-labeled antibody.

The AuNPs and AuNP-labeled antibody (Au NPs@PAb-1) were characterized using their UV–vis spectrum, as shown in Figure 2. The adsorption peak of AuNPs shifted from 521 nm to 532 nm, which indicated that the antibody successfully conjugated to the AuNPs.

**Figure 3:**
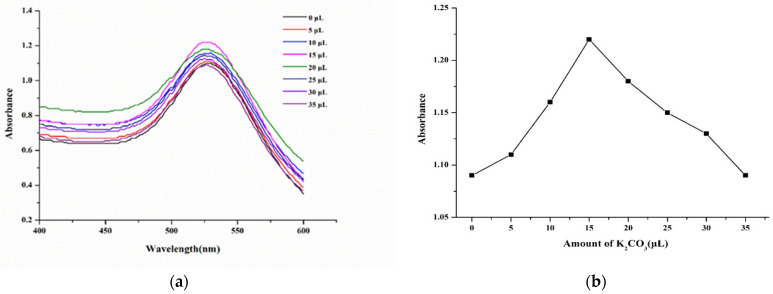
(**a**) Wavelength scanning of AuNP-labeled antibody with different amounts of K_2_CO_3_. (**b**) Absorbance of AuNP-labeled antibody with different amounts of K_2_CO_3_.

The combination of AuNPs and antibody mainly depended on the pH and the amount of binding antibody. When the pH of the system was close to the isoelectric point of the antibody protein, the binding between AuNPs and antibody was more stable. For 1 mL of AuNP solution, the pH value was adjusted by adding different amounts of a 0.2 M potassium carbonate (K_2_CO_3_) solution. As shown in Figure 3, the absorbance first increased and then decreased with an increasing amount of K_2_CO_3_ solution. When the volume was set to 15 μL, the solution absorbance of the AuNP-labeled antibody reached a maximum. Therefore, 15 μL of K_2_CO_3_ solution was used for adjusting the pH of 1 mL of AuNP solution.

**Figure 4:**
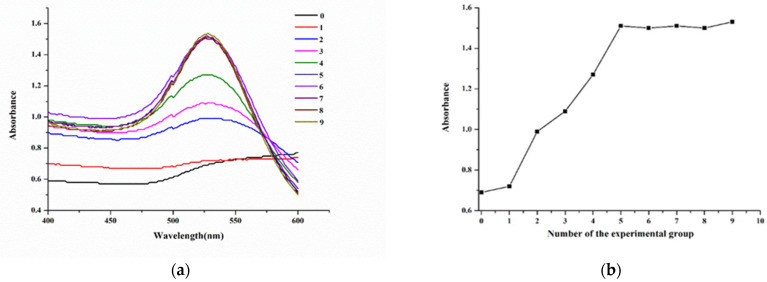
(**a**) Wavelength scanning of AuNP-labeled antibody with different amounts of antibody. (**b**) The absorbance of AuNP-labeled antibody with different amounts of antibody.

The amount of antibody used for labeling was also a key factor; an insufficient amount of antibody would result in the surface instability of AuNPs, while excess antibody would lead to the waste of antibody. According to Figure 4, when the antibody amount was 9.35 μg (experimental group 5 in Figure 4b), the amount of labeling antibody had already reached saturation. Based on these parameters, for 1 mL of AuNP solution, the optimum labeling conditions were set to 15 μL of 0.2 M K_2_CO_3_ solution for pH adjustment and 9.35 μg of antibody for conjugation.

### 2.3. Establishment of AuNP Lateral-Flow Immunochromatographic Strip Assay

**Figure 5:**
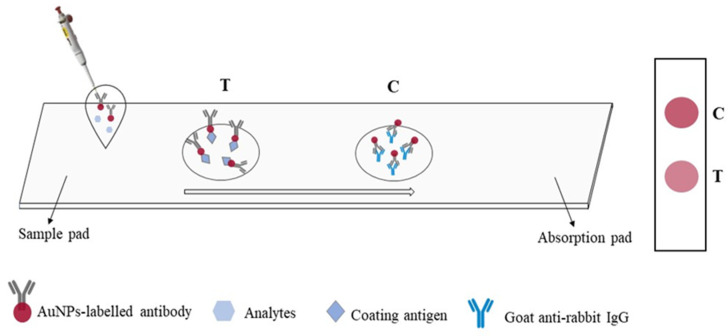
Schematic diagram of AuNPs lateral-flow immunoassay.

**Figure 6:**
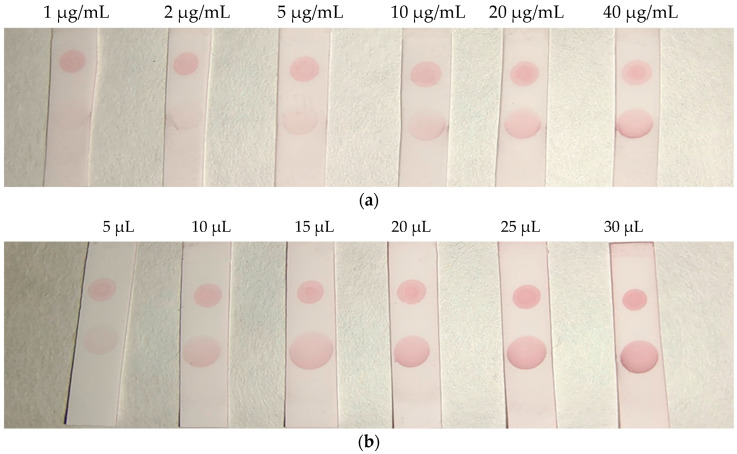
(**a**) Optimization of the coating antigen. (**b**) Optimization of AuNP-labeled antibody.

An immunochromatographic strip assay was developed on the basis of an antibody–antigen competitive immunoreaction. The process is depicted in Figure 5. The reaction principle is as follows: In the absence of BADGE in the sample, the AuNP-labeled antibody binds to the coating antigen at the test (T) zone, which turns red. This result is considered negative. Meanwhile, BADGE competes with the coating antigen (T zone) to bind the AuNP-labeled antibody, thereby weakening the color of the T zone. This color weakening is more pronounced as the BADGE concentration increases. The concentrations of the coating antigen and AuNP-labeled antibody in the lateral-flow immunoassay strip assay were optimized. As shown in Figure 6a, the color of the T zone gradually darkened with increasing concentration of the coating antigen up to 20 μg/mL. As shown in Figure 6b, when the volume of AuNP-labeled antibody reached 20 μL, the color of the T zone did not change considerably. Therefore, 20 μL of AuNP-labeled antibody solution was selected for the following test.

**Figure 7:**
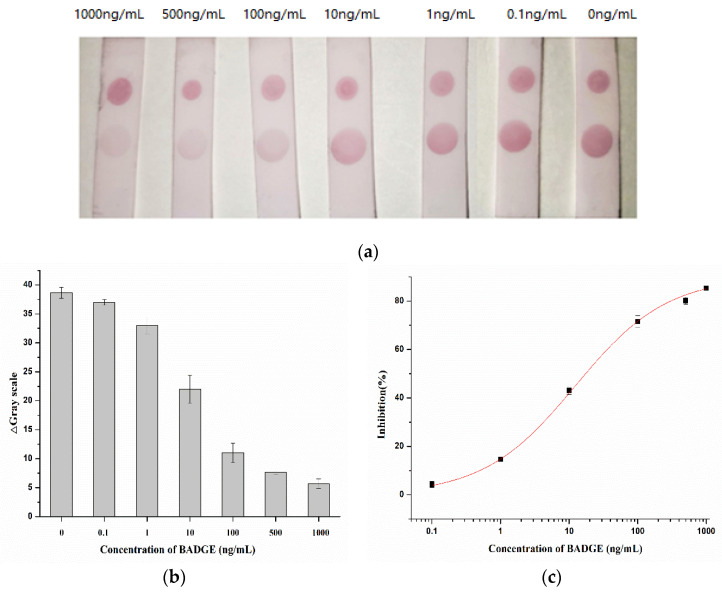
(**a**) Visual results of AuNPs lateral-flow immunoassay strip assay (the concentration of BADGE: 0, 0.1, 1, 10, 100, 500, 1000 ng/mL). (**b**) Grayscale variation in T zones for different concentrations of BADGE. (**c**) Inhibition analysis for BADGE.

Under optimal conditions, the AuNP-labeled antibody was mixed with different concentrations of BADGE and added to the strip. As shown in Figure 7a, the intensity of the color of the T zone decreased as the BADGE concentration increased. When the BADGE concentration reached 1 ng/mL, the color of the T zone could be distinguished from that of the negative sample (without BADGE); therefore, the visual detection limit was set to 1 ng/mL.

Color intensities were quantified using Adobe Photoshop CC software. The grayscale variation in the T zone and the standard curve drawn by calculating the inhibition ratio are shown in Figure 7b and 7c, respectively. The calculated limit of detection, which was defined as the concentration causing 15% inhibition (IC15), was 0.97 ng/mL. This result is consistent with the visual result.

### 2.4. Stability Analysis of AuNPs-PAb Immunochromatographic Strip

The test strips were stored at 4 °C for 1, 3, 5, and 7 days, and the color was observed via the immunochromatographic strip assay using a standard 1 ng/mL BADGE solution. The results are shown in Table 2. No remarkable change was observed in the T zone, indicating the good stability of the test strips for the detection of BADGE.

### 2.5. Matrix Effect

**Figure 8:**
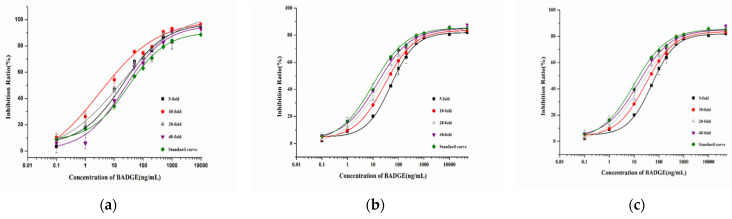
The optimized dilution of the sample-extracting solution: (**a**) canned luncheon meat; (**b**) canned yellow peach; (**c**) Red Bull drink (Hainan Red Bull Beverage Co., Ltd., Lingao, China).

In the analysis of real samples, the matrix can interfere with the detection accuracy. Therefore, to eliminate the matrix effect, the samples were diluted to different concentrations (5-, 10-, 20-, and 40-fold dilution) and analyzed via ic-ELISA. As shown in Figure 8, the 40-fold dilution could remove the matrix effect almost completely, enabling the analysis using the assay.

### 2.6. Sample Recovery Analysis

**Figure 9:**
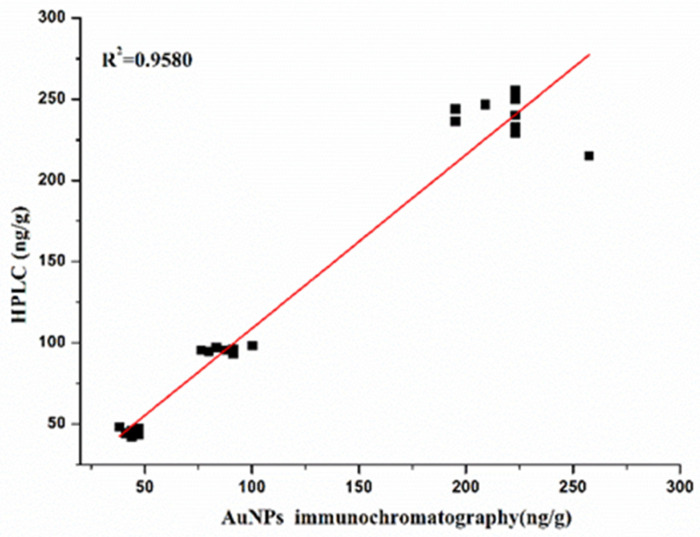
Correlation analysis between AuNPs lateral-flow immunochromatographic strip assay and HPLC.

Spiking and recovery analyses were performed using the AuNP-labeled antibody immunochromatographic strip assay, which was validated via HPLC analysis (Table 3). The recoveries of BADGE in canned food samples ranged from 79.86% to 93.81%. The correlation coefficient between the AuNP-based lateral-flow immunochromatographic strip assay and HPLC analysis was 0.9580 (Figure 9).

BADGE and its derivatives may exist simultaneously in real samples, thus the proposed immunochromatographic strip assay was used to detect the total amount of BADGE and its derivatives and validated via HPLC analysis. As shown in Table 4, the total amount of BADGE compounds in the samples determined using the immunochromatographic strip assay was consistent with that obtained via HPLC, demonstrating that the developed AuNP-based lateral-flow immunochromatographic assay could be used as a practical biological monitoring method for the rapid screening of BADGE and its derivatives.

## 3. Discussion

BADGE is widely used in the preparation of the inner coatings of metal cans. It is often used to avoid direct contact between the food matrix and metals in packaging materials. During processing, storage, and transportation, BADGE is prone to migrating into the food matrix and generating hydrated or chlorinated derivatives, which can accumulate in the human body through the food chain, causing abnormalities in the endocrine and nervous systems. Anna et al. [33] studied the toxicity and potential lipid destruction of BADGE in human placental JEG-3 cells and found that it can interfere with lipid metabolism and alter the cellular lipidome, ultimately causing disease. Instrumental analysis methods have been used to detect BADGE and its derivatives. For instance, Gallo et al. [34] simultaneously determined eight kinds of bisphenol substances, including BADGE, in soft drinks via liquid chromatography–fluorescence.

Due to the wide polarities of BADGE and its derivatives, conventional LC requires complex sample pretreatment to complete the separation, which cannot meet the need for rapid detection. Moreover, traditional instrumental analysis methods cannot realize the simple and rapid analysis of large quantities of samples or the real-time monitoring of migrated residual pollution.

In contrast, immunoassays based on antibodies or other biological molecules enable rapid analysis with simple pretreatments and constitute an effective supplementary method to conventional instrumental analysis methods. In fact, immunoassays have been widely used for the analysis of various pollutants in the field of food safety because of their reliability, efficiency, and cost savings. Accordingly, they have been recommended by institutional analysis departments for the rapid screening of large quantities of samples. Considering the health hazards posed by BADGE compounds, the monitoring of food via long-term, multifrequency, high-throughput sample analysis is required, for which immunoassays are suitable methods. Guan [35] developed a fluorescence polarization assay for the simultaneous monitoring of BPA, BPF, BADGE, and BFDGE in canned tuna with detection limits of 0.35, 0.08, 0.10, and 0.49 mg/L, respectively. The method was not sufficiently sensitive for the detection of BADGE, which may be because the receptor cannot completely replace the biological antibody to achieve high-affinity recognition of the target compound. The broad-spectrum polyclonal antibody used in this study can simultaneously recognize BADGE and its derivatives, enabling the development of a rapid AuNP-based lateral-flow immunochromatographic strip assay for the detection of BADGE and its hydrolyzed and chlorinated derivatives in canned food. The assay can be performed within 15 min. The visual results were processed using Adobe Photoshop CC software, and the detection limit (IC15) was 0.97 ng/mL. Therefore, this method can meet the need for real-time screening and the detection of large quantities of samples. The developed AuNPs-based lateral-flow immunochromatography assay is compared with other methods reported for the detection of BADGE and its derivatives in Table 5.

## 4. Materials and Methods

### 4.1. Chemicals and Reagents

Chloroauric acid (HAuCl_4_·4H_2_O), trisodium citrate dihydrate, sodium chloride (NaCl), K_2_CO_3_, disodium hydrogen phosphate dodecahydrate (Na_2_HPO_4_·12H_2_O), sodium phosphate dibasic dihydrate (Na_2_HPO_4_·2H_2_O), sulfuric acid (H_2_SO_4_), Tween-20, ethyl acetate, hexane, acetonitrile, and methanol were purchased from Sinopharm Chemical Reagent (Shanghai, China). The chemical standards BADGE, BADGE·H_2_O, BADGE·HCl, and BADGE·HCl·H_2_O were purchased from Macklin (Shanghai, China). Bovine serum albumin (BSA), nonfat milk powder, and goat anti-rabbit IgG-HRP were purchased from Sangon Biotech (Shanghai, China). Canned luncheon meat, canned yellow peach, and Red Bull drinks were purchased from a local supermarket (Zhenjiang, China).

### 4.2. Instruments

An HH-A magnetic stirrer was purchased from Zhongda Instrument Factory (Changzhou, China), and a UV-1801 ultraviolet spectrophotometer was purchased from Ruili Company (Beijing, China). A smartphone (Huawei P40 with a rear camera of 8 million pixels, Huawei Technologies Co., Ltd, Shenzhen, China) was used to take photos. HPLC was performed using an LC-20AD module (Shimadzu, Japan) coupled to an ultraviolet detector. A Hypersil GOLD C18 column (4.6 mm × 250 mm, 5 μm) was employed for chromatographic separation in the HPLC analysis. The mobile phase was composed of 40% ultrapure water and 60% acetonitrile. The eluent flow rate was 0.5 mL/min, the injection volume was 10 µL, and the temperature of the column oven was maintained at 30 °C.

### 4.3. Screening of Broad-Spectrum Antibodies

Four antisera with certain recognition abilities for BADGE and its derivatives were screened. The cross-reactivity of the four antisera, which was investigated to determine their broad-spectrum recognition specificity, was calculated as follows:

Cross-reactivity (%) = IC_50_ (BADGE)/IC_50_ (other analogs) × 100

### 4.4. Preparation of AuNPs

AuNPs were produced according to the sodium citrate method. Briefly, 100 mL of a 0.01% HAuCl_4_ solution was heated and kept boiling. Then, 4 mL of 1% sodium citrate was quickly added under stirring, and the resulting solution was heated and stirred for 5–10 min until the color was stable. The obtained AuNP solution was cooled to room temperature and stored at 4 °C.

### 4.5. Preparation of AuNP-Labeled Antibody

#### 4.5.1. Optimization of pH Value of AuNPs Solution

The pH value of the AuNP solution was adjusted by adding different amounts of K_2_CO_3_ solution. Excess amounts of antibodies were added and allowed to stand at room temperature for 10 min. Further, 10% NaCl was added and vortex-mixed. After standing for 10 min, the color was observed and scanned via UV–vis spectroscopy.

#### 4.5.2. Optimization of the Amounts of Antibody

As shown in Table 6, different amounts of antibody were added to the pH-adjusted AuNP solution, and the mixture was allowed to stand at room temperature for 10 min. Then, 10% NaCl was added and vortex-mixed. After standing for 10 min, the color was observed and scanned via UV–vis spectroscopy.

Overall, 10 mL of a AuNP solution was adjusted to the optimal pH value by adding a 0.2 M K_2_CO_3_ solution. The antibody was added to the resulting AuNP solution, and the mixture was allowed to react at 4 °C for 330 min. Then, 10% BSA was added to block the unbinding sites. The solution was centrifuged at 12,000 rpm for 20 min, and the precipitate was redissolved in 1 mL of PB solution containing 0.05% Tween-20 and 1% BSA. The resulting solution was stored at 4 °C until use.

### 4.6. Establishment of AuNPs Lateral-Flow Immunochromatographic Assay

A typical immunochromatographic test strip consisted of a sample pad, a conjugate or reagent pad, a capture zone (test line and control line), and an absorbent pad. A nitrocellulose membrane with smooth backing was used to prepare the immunochromatographic strips, which were cut to a certain size. The T zone was coated with the coating antigen, and the control (C) zone was coated with goat anti-rabbit IgG. The strip was then dried at room temperature and stored at 4 °C. A certain volume of AuNP-labeled antibody was mixed with 100 μL of a BADGE standard solution and incubated for 10 min. The mixture was added to the sample zone. After 15 min, the liquid completely flowed through the T and C zones. The visualized results were recorded using a smartphone (Huawei Technologies Co., Ltd., Shenzhen, China) and processed using Adobe Photoshop CC software for quantitative analysis. All photos were recorded during the daytime without a flashlight.

Inhibition was calculated using the following equation:

Inhibition (%) = (△G − △G1)/(△G) × 100%,

where G and G1 represent the grayscale variation without and with different concentrations of BADGE (or its derivatives), respectively.

### 4.7. Sample Analysis

Canned luncheon meat, canned yellow peach, and Red Bull drinks obtained from a local supermarket were selected as samples for analysis. The canned luncheon meat samples were treated as follows: 2.0 g of sample was homogenized with 10 mL hexane and subjected to ultrasonic-assisted extraction for 30 min. The mixture was centrifuged at 4500 rpm for 10 min. The supernatant was extracted twice using 5 mL of acetonitrile. The acetonitrile extracts were evaporated to dryness at 40 °C under nitrogen. Subsequently, the residues were redissolved in 2 mL of methanol. The canned yellow peach samples were treated as follows: 2.0 g of sample was weighed, and 5 mL of ethyl acetate was added as the extraction solvent. The mixture was shaken for 20 min in a shaker and then transferred to an ultrasonic bath for 30 min. The mixture was centrifuged at 4500 rpm for 15 min. The supernatant was evaporated to dryness under a nitrogen stream. Furthermore, the extract was redissolved in 2 mL of methanol. The Red Bull samples (5 mL) were centrifuged at 4500 rpm for 10 min, and the supernatant was filtered through a 0.22 μm membrane before analysis. The samples were separately spiked with BADGE at different concentrations (50, 100, and 250 ng/g) to evaluate the accuracy of the AuNP-based lateral-flow immunochromatographic strip assay, and the results were confirmed using HPLC.

## 5. Conclusions

A broad-spectrum polyclonal antibody that can recognize BADGE and its BADGE·HCl, BADGE·H_2_O, and BADGE·HCl·H_2_O derivatives was evaluated and coupled with AuNPs to develop a AuNP-based lateral-flow immunochromatographic strip assay for the simultaneous detection of BADGE and its derivatives in canned food. The visualized results were processed using Adobe Photoshop CC software and validated via HPLC analysis. The developed broad-spectrum lateral-flow immunochromatographic strip assay is reliable and accurate and meets the requirements for the rapid screening of large quantities of samples because of its simplicity, rapid operation, and cost effectiveness.

## Figures and Tables

**Table 1:** Cross-reactivities of antisera with BADGE, BADGE·HCl, BADGE·H_2_O, and BADGE·HCl·H_2_O.

Target	Antisera							
	PAb-1		PAb-2		PAb-3		PAb-4	
	IC_50_ (ng/mL)	CR (%)	IC_50_ (ng/mL)	CR (%)	IC_50_ (ng/mL)	CR (%)	IC_50_ (ng/mL)	CR (%)
BADGE	51	100	65	100	26	100	29	100
BADGE·HCl	64	79.6	114	57.0	57	45.6	60	48.3
BADGE·H_2_O	29	175.8	51	127.4	76	34.2	47	61.7
BADGE·HCl·H_2_O	46	110.8	59	110.1	82	31.7	70	41.4

**Table 2:** Stability analysis of AuNP-labeled antibody immunochromatographic strip assay.

1 (Day)	3 (Day)	5 (Day)	7 (Day)
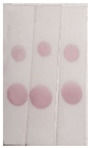	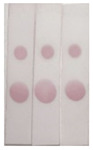	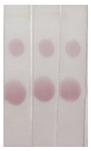	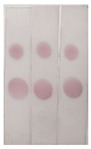

**Table 3:** Recoveries of BADGE and its derivatives in spiked samples by AuNPs lateral-flow immunochromatographic strip assay and HPLC (*n* = 3).

Sample	Spiked Conc. (ng/g)	HPLC (*n* = 3)		AuNPs Lateral-Flow Immunochromatographic Strip Assay (*n* = 3)		
		Mean ± SD (ng/g)	Recovery (%)	Mean ± SD (ng/g)		Recovery (%)
Canned luncheon meat	50	45.28 ± 0.02	90.56	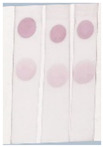	45.67 ± 1.66	91.35
	100	95.71 ± 0.10	95.71	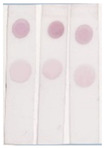	79.86 ± 3.51	79.86
	250	247.56 ± 0.11	99.02	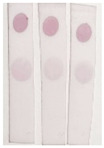	209.10 ± 5.59	83.64
Canned yellow peach	50	43.30 ± 0.03	86.60	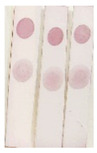	44.10 ± 2.60	88.20
	100	96.93 ± 0.09	96.93	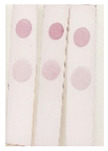	91.69 ± 6.94	91.69
	250	243.95 ± 0.74	97.58	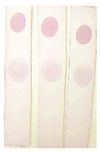	213.76 ± 5.27	85.50
Red Bull drink	50	46.97 ± 0.03	93.94	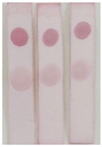	43.17 ± 3.78	86.35
	100	94.82 ± 0.11	94.82	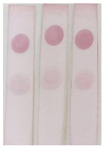	87.35 ± 3.97	87.35
	250	231.41 ± 0.50	92.56	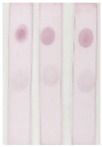	234.54 ± 6.48	93.81

**Table 4:** Detection of BADGE and its derivatives in samples using AuNPs lateral-flow immunochromatographic strip assay and HPLC (*n* = 3).

Sample		BADGE and Its Derivatives	HPLC		AuNPs Lateral-Flow Immunochromatographic Strip Assay		
			Mean ± SD (ng/g)	Total Amount	Detection of Concentration Mean ± SD (ng/g)		Actual Concentration Mean ± SD (ng/g)
Canned luncheon meat	1	BADGE	45.03 ± 0.83	130.06	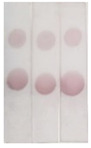	3.14 ± 5.44	125.72
		BADGE·HCl	85.03 ± 0.36				
		BADGE·H_2_O	ND				
		BADGE·HCl·H_2_O	ND				
	2	BADGE	39.00 ± 0.80	110.76	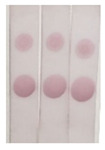	2.57 ± 1.09	103.69
		BADGE·HCl	71.76 ± 0.19				
		BADGE·H_2_O	ND				
		BADGE·HCl·H_2_O	ND				
Canned yellow peach	3	BADGE	47.85 ± 0.39	102.11	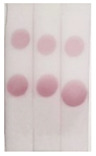	2.48 ± 0.84	99.42
		BADGE·HCl	ND				
		BADGE·H_2_O	54.25 ± 0.31				
		BADGE·HCl·H_2_O	ND				
	4	BADGE	61.39 ± 0.25	144.78	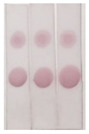	3.41 ± 1.72	136.50
		BADGE·HCl	ND				
		BADGE·H_2_O	83.42 ± 0.07				
		BADGE·HCl·H_2_O	ND				
Red Bull drink	5	BADGE	50.54 ± 0.77	176.92	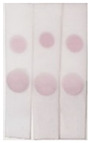	4.27 ± 2.64	170.81
		BADGE·HCl	ND				
		BADGE·H_2_O	126.37 ± 0.82				
		BADGE·HCl·H_2_O	ND				
	6	BADGE	56.49 ± 2.16	193.68	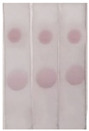	4.67 ± 5.44	187.14
		BADGE·HCl	ND				
		BADGE·H_2_O	137.18 ± 0.62				
		BADGE·HCl·H_2_O	ND				

**Table 5:** Comparison of analytical methods for the detection of bisphenol derivatives (including BADGE).

Detection Method	Detection Targets	Detection Results	References
HPLC-FLD	Bisphenols, bisphenol diglycidyl ethers and their derivatives	Quantitation limits for the analytes ranged between 0.9 and 3.5 μg kg^−1^	Ref. [16]
HPLC-FLD	5 bisphenol derivatives including BADGE	Limits of detection (LODs) were between 21 and 28 ng/mL	Ref. [17]
HPLC-FLD	Bisphenol A diglycidyl ether and its derivatives	LODs varied from 0.01 to 0.20 ng/g	Ref. [18]
LC–MS/MS	BPA and its derivatives including BADGE	Quantification limits were in the range of 2–10 μg kg^−1^	Ref. [19]
UHPLC-ESI-MS/MS	Bisphenol ethers and their derivatives	Limits of quantitation (LOQs) for the analytes ranged from 0.02 to 5 mg/kg	Ref. [21]
GC-MS	Bisphenols and their diglycidyl ethers	Migration of BPA is between 104.67 and 181.46 μg L^−1^	Ref. [22]
ic-ELISA	BADGE BADGE·HCl BADGE·H_2_O BADGE·HCl·H_2_O	IC_15_ of BADGE, BADGE·H_2_O, BADGE·HCl, BADGE·HCl·H_2_O were 0.73, 0.39, 0.78, 1.45 ng/mL	Ref. [36]
AuNPs-based immunochromatographic strip assay	BADGE BADGE·HCl BADGE·H_2_O BADGE·HCl·H_2_O	Simultaneous detection of BADGE and its derivatives within 15 min. Visual detection limit was 1 ng/mL for BADGE.	This work

**Table 6:** pH-adjusted AuNPs solution for labeling different amounts of antibody.

Number of Experimental Group	0	1	2	3	4	5	6	7	8	9
Antibody (μg)	0	0.37	1.49	3.36	5.98	9.35	13.51	18.33	23.93	30.32
AuNPs solution (mL)	1	1	1	1	1	1	1	1	1	1

## Data Availability

Data are contained within the article.
